# Splenic B-Cell Lymphomas with Diffuse Cyclin D1 Protein Expression and Increased Prolymphocytic Cells: A Previously Unrecognized Diagnostic Pitfall

**DOI:** 10.1155/2018/5761953

**Published:** 2018-09-27

**Authors:** Khaled Algashaamy, Yaohong Tan, Nicolas Mackrides, Alvaro Alencar, Jing-Hong Peng, Joseph Rosenblatt, Juan P Alderuccio, Izidore S. Lossos, Francisco Vega, Jennifer Chapman

**Affiliations:** ^1^Department of Pathology, Division of Hematopathology, University of Miami, Sylvester Comprehensive Cancer Center and Jackson Memorial Hospital, Miami, Florida, USA; ^2^Department of Medicine, Division of Hematology-Oncology, University of Miami, Sylvester Comprehensive Cancer Center and Jackson Memorial Hospital, Miami, Florida, USA

## Abstract

Prolymphocytic transformation is a concept usually applied in the context of chronic lymphocytic leukemia/small lymphocytic lymphoma to describe the presence of a high percentage of prolymphocytes in peripheral blood (usually more than 55%). Prolymphocytic transformation has also been reported in mantle cell lymphoma (MCL) but only rarely in splenic marginal zone lymphoma (SMZL). We present two splenic B-cell lymphomas presenting in the leukemic phase and with increased prolymphocytes, both classified as SMZL with prolymphocytic transformation. One case clinically simulated B-prolymphocytic leukemia (B-PLL). Both lymphomas were very unusual because the tumor cells diffusely and strongly expressed cyclin D1 despite lacking the t(11; 14)(q13; q32) as detected by several approaches including next-generation sequencing, fluorescence *in situ* hybridization using *CCND1* break apart probe and fusion probes for t(11; 14)(q13; q32), and conventional karyotyping. These cases therefore simulated prolymphocytic variants of MCL. The incidence of this phenomenon is unknown, and awareness of this potential alternate protein expression pattern is important in order to avoid diagnostic errors.

## 1. Introduction

Splenic marginal zone lymphoma (SMZL) is an indolent, extranodal mature B-cell lymphoma typically presenting with massive splenomegaly and bone marrow and peripheral blood involvement [1, 2]. Neoplastic lymphoid cells are predominantly small, mature B cells with abundant pale cytoplasm. In the spleen, the tumor cells involve the white pulp in a micronodular pattern, invade the red pulp, and exhibit marginal zone differentiation [1]. SMZL are most frequently CD20 positive B-cell lymphomas that lack expression of CD5, CD10, CD43, CD23, and cyclin D1. Scattered large lymphoid cells are typical, particularly within the marginal zone of involved follicles. Additional features of SMZL include villous lymphocytes in the peripheral blood, intrasinusoidal infiltration of the bone marrow and, in a subset of cases, allelic loss of chromosome 7q22-36 [3].

SMZL is clinically characterized by a relatively indolent disease course unless adverse prognostic factors are present, such as poor performance status, high tumor burden, *TP53* abnormality, 7q deletion, and/or absence of *IgVH* somatic mutation [4]. A subset of SMZLs (∼5–13%) progresses to diffuse large B-cell lymphoma (DLBCL), which can be identified at the time of initial diagnosis or years later [5–7]. The rate of histologic transformation of SMZL to DLBCL is slightly higher than that of extranodal MZL and chronic lymphocytic leukemia/small lymphocytic lymphoma (CLL/SLL) (1–10%) but is less frequent than that of follicular lymphoma (25–60%). Although mantle cell lymphoma (MCL) does not undergo transformation to DLBCL per se, histologic progression to blastoid or pleomorphic variants can occur (11–39% of cases) [5, 6]. The mechanisms underlying histologic transformation of SMZL are poorly understood, but it is clear that this event is clinically relevant given its association with shorter time to progression and overall survival [7]. SMZLs have been described to have high genomic instability, in part related to loss of the *POT1* gene (at 7q31.32), which may contribute to progression to large cell lymphoma [3, 8].

As opposed to that in CLL/SLL and MCL, prolymphocytic transformation of SMZL is infrequently reported. One series of splenic lymphomas showing prolymphocytic transformation as defined by increased prolymphocytes in peripheral blood included 4 cases, 3 of which were classified as SMZL [9]. In this series, patients were elderly, presented with splenomegaly, marrow involvement, and leukocytosis with >55% prolymphocytes in peripheral blood. All cases in this previous report were negative for cyclin D1 protein. Deletion 7q was present in 3 of the cases, supporting the concept that this deletion is a risk factor for histologic transformation in the context of SMZL.

Similar to SMZL, B-cell prolymphocytic leukemia (B-PLL) is also a mature B-cell chronic leukemia presenting with peripheral blood, spleen, and bone marrow involvement. However, as opposed to SMZL, lymphoma cells with prolymphocytic morphologic features comprise >55% of tumor cells in peripheral blood, and patients typically present with B symptoms, massive splenomegaly, and markedly elevated white blood cell counts, usually >100 × 10^9^/L [1]. Similar to SMZL, B-PLL tumor cells are CD20 positive and usually negative for CD5, CD10, CD23, and cyclin D1. Rates of CD5 and CD23 expression in B-PLL are higher than those in SMZL with approximately 20–30% of B-PLL expressing CD5 and 10–20% expressing CD23 [1]. While initial reports of cytogenetic abnormalities in B-PLL identified a high rate of *IgH*/*CCND1* translocations, cases with this translocation are now classified as MCL. B-PLL lacking *IgH*/*CCND1* translocations frequently have complex karyotypes, but specific cytogenetic abnormalities are not known. Deletions at 17p13 and 13q14 are relatively frequent as are *TP53* mutations [1].

In this report, we present two unusual cases of non-MCL splenic B-cell leukemia/lymphoma that underwent prolymphocytic transformation or presented with increased prolymphocytes and showed diffuse cyclin D1 protein expression. These cases therefore simulated the prolymphocytic variant of MCL and represented an important diagnostic pitfall. To our knowledge, diffuse cyclin D1 protein expression has not been previously reported in SMZL or in its prolymphocytic transformation.

## 2. Case Presentations

### 2.1. Case 1

#### 2.1.1. Clinical History

This patient was a 71-year-old male who presented with abdominal discomfort and was found to have significant splenomegaly upon physical exam, confirmed by imaging. He was diagnosed clinically with SMZL and underwent splenectomy to control his disease. We have not been able to determine whether he had peripheral blood lymphocytosis at that time of initial presentation.

Ten years after the initial diagnosis of lymphoma, he presented to our institution with weight loss and extensive anterior mediastinal, hilar, and retroperitoneal lymphadenopathy with an anterior mediastinal mass measuring 9.8 × 5.6 cm. Serum lactate dehydrogenase was elevated (281 U/L, reference range: 135–225 U/L). He was started on weekly rituximab with no response. In view of progressive symptoms, he was switched to R-CHOP (rituximab, cyclophosphamide, doxorubicin, vincristine, and prednisone) therapy at an outside institution. He presented to our institution after his first cycle of R-CHOP for additional recommendations. He felt dramatically better at that time. The clinical impression was that he had a large-cell transformation of his previously diagnosed low-grade SMZL.

#### 2.1.2. Histopathologic, Immunophenotypic, and Molecular Findings

Laboratory analysis showed a white blood cell count of 9.4 × 10^3^ cells/*μ*L, hemoglobin level of 12.2 g/dL, platelet count of 242 × 10^3^/*μ*L, and absolute lymphocyte count of 1.33 × 103/*μ*L. Peripheral smear was morphologically unremarkable. Peripheral blood flow cytometry showed a minor monotypic B-cell population, CD5, CD10, and CD23 negative, comprising 0.7% of analyzed events, consistent with minimal peripheral blood involvement by his previously diagnosed lymphoma. He was referred for lymph node biopsy to clarify the diagnosis.

**Figure 1:**
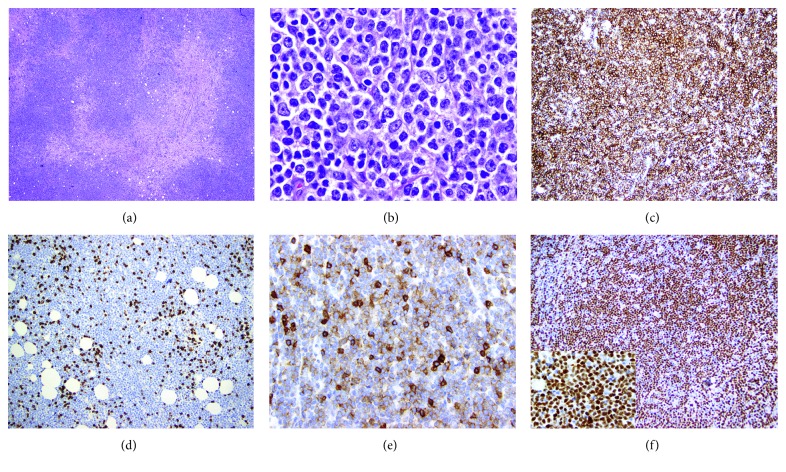
Case 1: lymph node biopsy showing relapsed splenic marginal zone lymphoma with prolymphocytic transformation and acquired CD5 (dim) and cyclin D1 (diffuse, strong) expression. Lymphoma cells consist of both small lymphoid cells with condensed nuclear chromatin, nuclear membrane irregularities, and monocytoid features and transformed cells with prolymphocytic features including intermediate to large size, more abundant cytoplasm, and prominent nucleoli (a, b). Lymphoma cells expressed CD20 (c), were negative for CD3 (d), a subset expressed dim CD5 (e), and cyclin D1 expression was diffuse and strong (f). (a and b) Hematoxylin and eosin staining, (a) 100 times magnification, (b) 500 times magnification, (c, d, and f) 200 times magnification, (e) 400 times magnification, and inset in (f) 1000 times magnification.

Excisional biopsy of the axillary lymph node showed extensive involvement by lymphoma composed of both small, cytologically atypical lymphoid cells with abundant cytoplasm admixed with more predominant intermediate to large-sized lymphoma cells with variably abundant cytoplasm, large nuclei, and prominent, centrally located nucleoli, consistent with prolymphocytes (Figures 1 and 1). The aggregates of prolymphocytes did not resemble proliferation centers seen in CLL/SLL as they were more discrete, monotonous, and expansile in nature. Transformed cells, defined as having intermediate to large-sized nuclei and central nucleoli, comprised more than 50% of all lymphoma cells. Sheets of large cells were not present; thus, designation as a large-cell transformation was not warranted [1, 10].

By immunohistochemistry, lymphoma cells were positive for CD20, CD5 (dim, subset), BCL2, and cyclin D1 (diffuse, positive in small lymphoma cells and those with prolymphocytic features), and negative for CD3, CD10, LEF1, and SOX11 (Figures 1–1). EBER staining by *in situ* hybridization was negative. The KI67 proliferation index was estimated at 40%, and it highlighted the large transformed cells in particular. Based on these findings, the possibility of mantle cell lymphoma (MCL) was considered.

**Figure 2:**
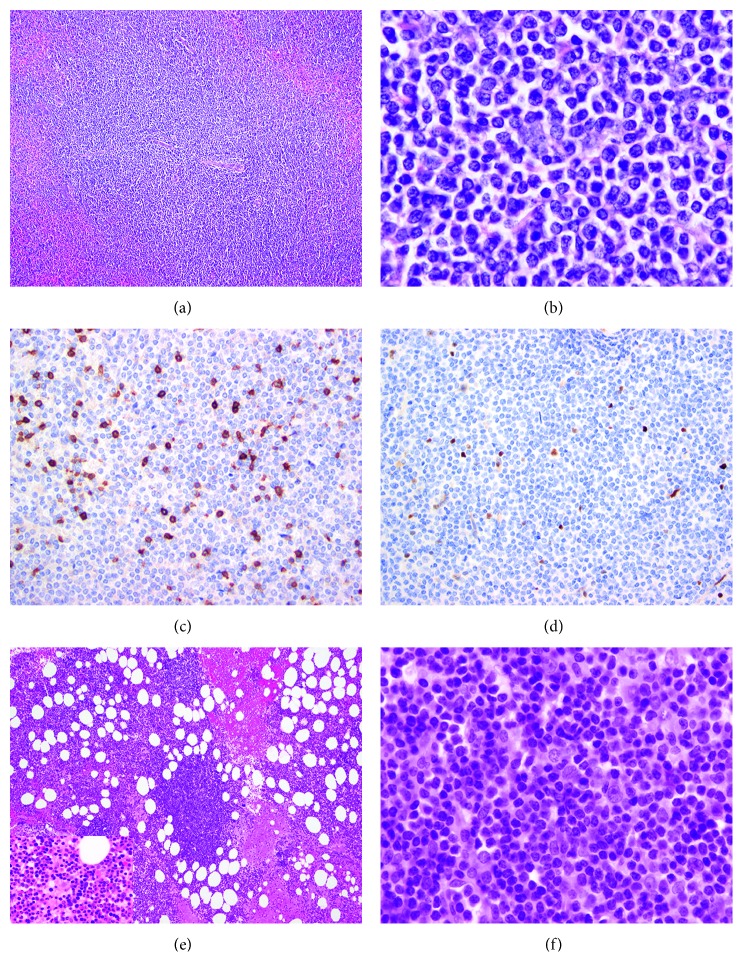
Case 1: splenic marginal zone lymphoma and staging bone marrow biopsy. Lymphoma cells are seen in a micronodular distribution expanding splenic white pulp and infiltrating adjacent red pulp (a) and are small with monocytoid morphologic features and minimal cytologic atypia (b). Tumor cells expressed CD20 (not shown) and were negative for CD5 and cyclin D1 (c and d). Bone marrow staging biopsy showed extensive involvement by lymphoma present predominantly in an interstitial nodular distribution with a focal sinusoidal pattern of involvement (inset in (e) showing focal sinusoidal distribution). Lymphoma cells were small in size and prolymphocytic or large cell transformation was not seen (f). (a, b, e, and f) Hematoxylin and eosin stain, (a and e) 100 times magnification, (b and f) 500 times magnification; CD5 (c) and cyclin D1 (d) immunohistochemistry stains shown at 400 and 200 times magnification, respectively.

The previous material (splenectomy and bone marrow biopsy from 10 years before) was then reviewed at our institution, and the original interpretation of SMZL was indeed confirmed. In the splenic resection, the lymphoma cells were present in a micronodular pattern within white pulp, invaded red pulp, and were composed of small-sized lymphoma cells with monocytoid morphologic features, negative for CD5 and cyclin D1 (Figure 2). Staging bone marrow biopsy performed at the time of initial splenectomy showed extensive involvement by low-grade lymphoma with an interstitial nodular and focally intrasinuoidal pattern (Figure 2). Lymphoid cells in the aspirate smear were small and did not have features of large-cell or prolymphocytic transformation. Karyotype and FISH studies performed in the bone marrow were negative for t(11; 14)(*CCND1-IgH*).

**Figure 3:**
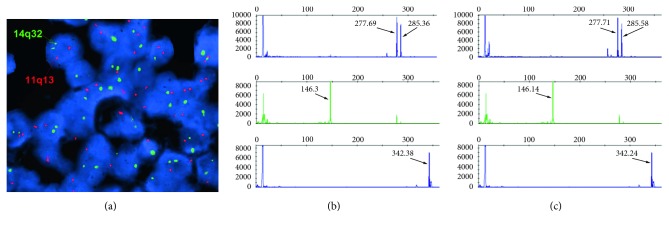
Case 1: FISH studies for *IgH*-*CCND1* fusion were negative (a). This assay was repeated in a reference lab and was again negative. PCR analysis was performed in the original splenic marginal zone lymphoma (b), and in the relapsed lymphoma (lymph node, and clonal PCR amplification products (c) were compared. Amplification products of the same size in base pairs in the initial and relapsed lymphomas were present, supporting that the tumors were clonally related.

Given the confirmation of the initially diagnosed SMZL, we performed FISH for t(11; 14)(*CCND1*-*IgH*) using *IgH* and *CCND1* dual-labeled probes in the lymph node sample both at our institution as well as at an external reference lab, and both were negative for *CCND1*-*IgH* rearrangement (Figure 3). An additional FISH test using a *CCND1* break apart probe was also performed and failed to detect rearrangements involving *CCND1*. Additional copies of *CCND1* were not present.

To assess the clonal relationship between the splenic and nodal neoplasms, PCR analysis for *IgH* rearrangement was performed in both samples. The same monoclonal *IgH* peaks (framework region (FR) 1: splenectomy: 342.24, lymph node: 342.38; FR2: splenectomy: 277.71, 285.58; lymph node: 277.69 and 285.36; FR3: splenectomy: 146.14, lymph node: 146.3, Figures 3 and 3) were identified in the original and current samples, supporting that the two lymphomas were clonally related. Next-generation sequencing (NGS) using the FoundationOne Heme comprehensive genomic profiling assay (Foundation Medicine, https://www.foundationmedicine.com/genomic-testing/foundation-one-heme) identified genomic alterations of *NOTCH2*, *BRCA2*, and *SRSF2* but was negative for rearrangements involving *CCND1* in both samples from the two different time points, further supporting that the lymphomas were clonally related and that despite the acquired strong expression of cyclin D1, the neoplasm lacked *CCND1* gene rearrangement or mutation.

Taken together, these findings indicate that the lymphoma with prolymphocytic transformation involving the lymph node represented disease progression of the patient's original SMZL. The prolymphocytic histologic transformation was defined in this case by the presence of sheets of intermediate to large-sized lymphoma cells with prolymphocytic morphologic features and was associated with acquired partial CD5 and diffuse cyclin D1 expression in the absence of acquired t(11; 14) (*CCND1*-*IgH*).

### 2.2. Case 2

#### 2.2.1. Clinical History

**Figure 4:**
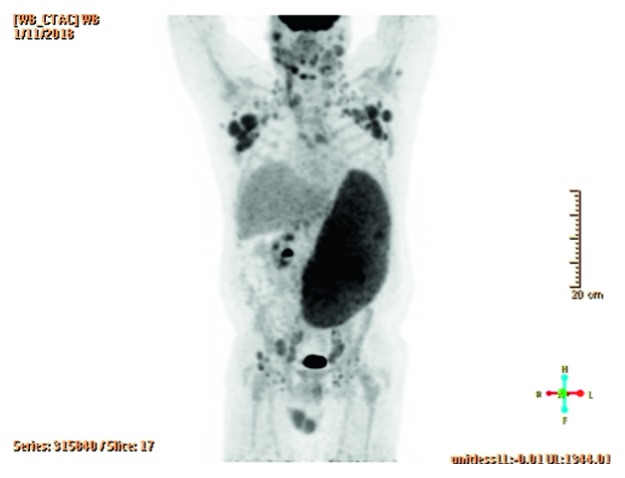
Case 2: PET imaging study confirmed massive splenomegaly, spleen measuring 31 cm in craniocaudal dimension, and with diffusely hypermetabolic activity, SUV 5.6, considered to be in keeping with lymphomatous involvement. There were also numerous subcapsular wedge-shaped areas of photopenia and hypodensities measuring up to 3.5 cm, which were suspected to be splenic infarcts.

This patient was a 53-year-old male without relevant past medical history who presented with one week of intermittent fevers, night sweats, weight loss, early satiety, cough, and exertional shortness of breath. Physical examination revealed diffuse small lymphadenopathy and massive splenomegaly. Imaging studies confirmed massive splenomegaly with the spleen measuring 31 cm in craniocaudal dimension with diffuse hypermetabolic activity, SUV 5.6, in keeping with lymphomatous involvement. There were also numerous subcapsular wedge-shaped areas of photopenia and hypodensities measuring up to 3.5 cm, which were suspected to be splenic infarcts (Figure 4).

#### 2.2.2. Histopathologic, Immunophenotypic, and Molecular Findings

**Figure 5:**
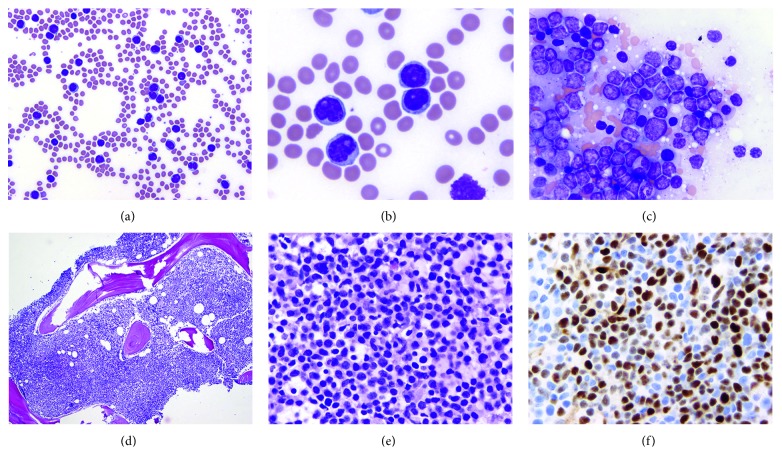
Case 2: peripheral blood and bone marrow biopsy. Wright Giemsa-stained peripheral blood smear shows lymphocytosis with many of the circulating lymphoma cells being small in size with round nuclear contours and minimal cytoplasm (a) 500x magnification. Approximately 50% of circulating lymphoma cells had more abundant cytoplasm and prominent nucleoli, consistent with prolymphocytic morphologic features (b) 1000x magnification. Bone marrow aspirate smear shows extensive involvement by lymphoma with a predominance of cells having prolymphocytic features (c 1000x magnification). Hematoxylin and eosin-stained slides of bone marrow core biopsy show extensive marrow involvement by lymphoma ((d) 200x and (e) 500x magnification) with lymphoma cells being diffusely positive for cyclin D1 protein (f) 500x magnification.

Laboratory analysis showed an elevated LDH of 421 U/L (normal range: 132–225 U/L), leukocytosis (white blood count: 210 × 10^9^ cells/L), anemia, and thrombocytopenia. Peripheral blood smear confirmed lymphocytosis with many circulating lymphoma cells being small to intermediate in size with mature nuclear chromatin (Figure 5). Approximately 50% of circulating lymphoma cells were large with abundant cytoplasm, more open and vesicular nuclear chromatin and prominent nucleoli, consistent with prolymphocytes (Figure 5). Cells with villous or circumferential cytoplasmic projections were not seen. Flow cytometry immunophenotyping in peripheral blood showed that lymphoma cells were positive for CD20, CD19, CD79a, CD22, and CD23 with lambda surface light chain restriction and negative for TdT, CD34, CD10, and CD5. Initial diagnostic considerations included prolymphocytic transformation of atypical CD5 negative CLL, B-prolymphocytic leukemia (B-PLL), and leukemic MCL, noting that the immunophenotypic expression patterns of B-PLL and SMZL can be indistinguishable.

Bone marrow core biopsy showed diffuse infiltration by intermediate-sized cytologically atypical lymphoma cells, and aspirate smear showed that most lymphoma cells had prolymphocytic morphologic features in the bone marrow (Figures 5–5). An intrasinusoidal pattern of involvement was difficult to appreciate due to the extensive degree of marrow involvement. Immunohistochemistry in the bone marrow core biopsy showed lymphoma cells were diffusely and strongly positive for cyclin D1 (Figure 5) and negative for CD5, LEF1, and SOX11. Chromosome analysis in bone marrow aspirate showed a normal karyotype 46, XY in 20 metaphases. FISH studies for t(11; 14) (*CCND1*-*IgH*) in the peripheral blood and bone marrow aspirate were negative but were positive for deletion 7q (33% of cells), deletion 17p (97% of cells), and deletion 13q (18% of cells). Next-generation sequencing using the FoundationOne Heme comprehensive genomic profiling assay identified a genomic alteration of *TP53* and was negative for rearrangements or mutations involving *CCND1* and other tested genomic alterations. Extra copies of *CCND1* were not detected.

Based on the absence of t(11; 14)(*CCND1*-*IgH*) as detected by FISH, karyotype, and NGS, a diagnosis of MCL was excluded despite diffuse cyclin D1 expression. The presence of massive splenomegaly, the lymphoma cell morphology and immunophenotype, and presence of deletion 7q, support that this lymphoma is best classified as SMZL with prolymphocytic transformation and diffuse cyclin D1 expression. However, extreme leukocytosis, presenting with B symptoms and diffuse lymphadenopathy, as seen in this case, is unusual for SMZL; thus, we cannot exclude that this lymphoma is a B-PLL with diffuse cyclin D1 expression. The presence of deletions 13q and 17p, although not specific, are recurrent abnormalities seen in approximately 27% and 50% of B-PLL, respectively, and may support this classification [1].

Because this patient did not have splenectomy, we also cannot completely exclude the possibility of splenic diffuse red pulp small B-cell lymphoma, although *CCND3* mutations, which are recurrent in that lymphoma, were not identified, and the presentation was more aggressive than typically reported in diffuse red pulp small B-cell lymphoma.

## 3. Discussion

We present two chronic B-cell leukemias with splenic involvement, both with increased prolymphocytes and showing misleading diffuse cyclin D1 protein expression. Further confounding the interpretations was that both leukemias were associated with aggressive clinical features and diffuse lymphadenopathy, clinically simulating MCL.

It is concerning that each of these cases would have been classified as MCL had the patients not had clinical scenarios drawing attention to the possibility of misleading cyclin D1 protein expression. Because FISH is not routinely performed in clinical cases to confirm MCL if diffuse cyclin D1 protein expression is present, it is possible that rare cases are misclassified.

Regarding the first case presented, we were able to identify that this was not an MCL because we were aware that the patient had a history of SMZL in which FISH studies were negative for t(11; 14)(q13; q32). FISH and NGS studies pursued in the relapsed, transformed lymphoma confirmed the lack of t(11; 14)(q13; q31) and variant translocations despite the acquired diffuse cyclin D1 protein expression. Supporting the classification of SMZL expressing cyclin D1 in this case was the initially indolent clinical presentation with splenomegaly and marrow involvement, initial lack of CD5 and cyclin D1 protein expression, lack of *CCND1*-*IgH*, and identification of *NOTCH2* mutation. Of note, we are able to conclude in this case that the expression of CD5 and cyclin D1 were acquired at or before the time of prolymphocytic transformation, but after initial presentation, given that the previous SMZL (CD5 negative and cyclin D1 negative) and current transformed SMZL (CD5 positive and cyclin D1 positive) were clonally related.

While not absolutely specific and also reported in diffuse large B-cell lymphomas and rarely in other low-grade B-cell lymphomas, activating *NOTCH2* genomic alterations are particularly frequent and recurrent in SMZL where they are observed in ∼20–25% of patients, establishing this gene mutation as one of the most frequent in SMZL [11, 12]. Due to the relative specificity of activating *NOTCH2* mutations, this abnormality informs the diagnosis of SMZL and distinguishes it from histopathologic mimics [11]. The prognostic significance of this mutation in the context of SMZL is controversial, but most studies associate this abnormality with poorer overall survival [4, 11, 13]. Analysis of *NOTCH2* mutations in SMZLs has shown a relationship with other alterations involved in lymphoma pathogenesis including deletion of 7q31, over-representation of IgHV1-2 usage, and higher promoter methylation status and correlates with poor outcome and histologic transformation [4, 13].

In addition to *NOTCH2* mutation, *BRCA2* mutation was also identified in case 1. *BRCA2* is a tumor suppressor gene whose protein regulates response to DNA damage and whose mutation causes the inability to repair DNA damage, thereby promoting tumorigenesis [14].

While *BRCA2* mutations are reported in DLBCL, we are not aware that this mutation has been reported specifically in SMZL [15]. Similarly, *SRSF2* mutation, also identified in this case, is widely reported in myeloid neoplasms, but we are not aware of its significance in MZL [16].

The second case presented is another example of a diffuse cyclin D1 positive B-cell lymphoma presenting initially in the leukemic phase and with increased prolymphocytes (50%), diffuse lymphadenopathy, massive splenomegaly, and aggressive clinical features. Despite diffuse cyclin D1 protein expression, FISH, karyotype and NGS did not identify alterations of *CCND1*. Deletions of 7q, 17p, and 13q were identified, and *TP53* mutation was detected by NGS. While not diagnostically specific, deletion 13q is reported in B-PLL, and 17p deletions and *TP53* mutations are particularly frequent in B-PLL (∼50% of cases). Given these genetic abnormalities and the clinical presentation, we cannot exclude that this lymphoma is a B-PLL presenting initially with diffuse cyclin D1 protein expression.

However, also present in this case was a 7q deletion, which, although also not specific, is reported in and supports the classification of this lymphoma as SMZL with leukemic prolymphocytic transformation. In fact, similar cases of SMZL with the overt leukemic phase including increased peripheral blood prolymphocytes (>55%), markedly elevated leukocyte count (>130 × 10^9^/L), and massive splenectomy are previously reported [9]. Similar to our case, these previously reported SMZL with prolymphocytic transformation presented with a prolymphocytic leukemia-like clinical picture, and the resected spleens showed SMZL with increased nucleoliated cells consistent with prolymphocytes. Deletion 7q was identified in these previously reported cases, supporting the diagnosis of SMZL with prolymphocytic transformation, overt leukocytosis, and a clinical picture mimicking B-PLL.

While SMZL lacks recurrent chromosomal translocations that are typical of other lymphomas, including other MZLs, deletion 7q, the most frequent copy number alteration, is identified in 30% of SMZL, and its presence supports the classification of this lymphoma type [17]. Although deletion 7q is not entirely specific for SMZL, it is seen only rarely in other small and leukemic B-cell lymphomas including chronic lymphocytic leukemia, hairy cell leukemia, and MCL. This deletion is not reported as a recurrent abnormality in B-PLL. In the context of SMZL, deletion 7q is associated with poor clinical outcomes [4, 13].

The target genes lost in the 7q deletion responsible for development of a subset of SMZL have not been clearly identified. Studies aimed at elucidating the oncogenic nature of the 7q deletion have shown that the deleted region in some cases is a 2.8 Mb region at 7q32 [17].

Recurrent breakpoints in this region have not been identified [17]. Loss of the sonic hedgehog gene (*SHH*) at 7q36.2 and protection of telomere 1 gene (*POT1*) at 7q31.32 have been reported in array-based comparative genomic hybridization studies aimed at characterization of the deletion 7q abnormality of SMZL [8]. It has been postulated that loss of *POT1* in particular may contribute to lymphomagenesis and create an environment of DNA instability, correlating with the poor prognostic significance of this deletion [8].

Unlike previously reported SMZL (with or without prolymphocytic transformation), our report describes diffuse cyclin D1 protein expression in SMZL, which has not been reported previously to our knowledge [18]. This is an important finding given that cyclin D1 protein expression is routinely used in clinical practice to identify leukemias/lymphomas as MCL. In our two cases, the diffuse expression of cyclin D1 was associated with the presence of a prolymphocytic transformation although both small and large (prolymphocytic) cells expressed cyclin D1.

We were able to identify one case report describing cyclin D1 positive marginal zone lymphoma of mediastinum; however, the provided image in this report shows only weak and focal nuclear positivity for cyclin D1 and is not the staining pattern or intensity expected for MCL [19]. Previous reports of SMZL in prolymphocytic transformation in particular have shown all cases to be negative for cyclin D1 protein [9].

*CCND1* is an oncogene that promotes progression of the cell cycle at the level of the G1 checkpoint, where its protein binds to CDK4 and CDK6 creating complexes that phosphorylate retinoblastoma protein, inactivating its suppressor effect [20]. *Cyclin D1* overexpression in the context of lymphomagenesis is most frequently due to a translocation that juxtaposes the 11q13 band containing *cyclin D1* with the constitutively active *IgH* locus on chromosome 14. In the context of B-cell lymphomas, the presence of this translocation and the resulting diffuse overexpression of cyclin D1 protein, detected by immunophenotyping, have come to define MCL. However, expression of *CCND1* can be modulated at the transcriptional level by other mechanisms including being promoted by transcription factors such as STAT3, independent of the t(11; 14)(q13; q32) translocation [21].

In the cases reported here, cyclin D1 protein expression was upregulated independent of chromosomal translocation or additional *CCND1* copies, neither of which were not detectable by a variety of means. The mechanism of protein overexpression in the cases we present is unclear at this time but is not unique to the lymphomas we report given that overexpression of cyclin D1 is known to occur in other nontranslocated lymphomas including diffuse large B-cell lymphoma and within proliferation centers of CLL/SLL. It is intriguing that cyclin D1 overexpression in our report was associated with prolymphocytic transformation, as is the precedent for this occurrence within prolymphocytes of CLL/SLL and in a subset of diffuse large B-cell lymphoma.

Given our experience of diffuse cyclin D1 expression in SMZL coupled with the fact that FISH studies are not performed in the majority of lymphomas classified as MCL, we suggest the possibility that a subset of splenic and/or leukemic lymphomas classified as MCL based on cyclin D1 expression may in fact be SMZL expressing cyclin D1. This may particularly be the case when increased large cells or prolymphocytic cells are present. Given that diffuse cyclin D1 positive SMZL is not previously reported, the incidence of this finding and its clinical significance are unknown and certainly need to be investigated. The association of prolymphocytic transformation and acquired cyclin D1 protein expression likewise deserves additional investigation. Finally, the cases presented also raise the question of whether FISH studies should be performed in cases of cyclin D1 positive B-cell lymphomas in order to confirm the diagnosis of MCL. We are not doing this in our practice despite acknowledgement of the cases presented.
